# Defining self‐monitoring in postpandemic maternity care: Perspectives from women, partners, healthcare professionals, and policymakers

**DOI:** 10.1111/aogs.70048

**Published:** 2025-09-05

**Authors:** Tisha Dasgupta, Harriet Boulding, Abigail Easter, Gillian Horgan, Hiten D. Mistry, Neelam Heera, Aricca D. Van Citters, Eugene C. Nelson, Peter von Dadelszen, The RESILIENT Study Group, Laura A. Magee, Debra E. Bick, Harriet Boulding, Kathryn Dalrymple, Tisha Dasgupta, Emma L. Duncan, Abigail Easter, Julia Fox‐Rushby, Gillian Horgan, Asma Khalil, Alice McGreevy, Hiten D. Mistry, Eugene C. Nelson, Lucilla Poston, Paul Seed, Sergio A. Silverio, Marina Soley‐Bori, Florence Tydeman, Aricca D. Van Citters, Sara White, Ingrid Wolfe, Yanzhong Wang, Peter von Dadelszen, Laura A. Magee, Sergio A. Silverio

**Affiliations:** ^1^ Department of Women & Children's Health, School of Life Course & Population Sciences King's College London London UK; ^2^ The Policy Institute, Faculty of Social Science & Public Policy King's College London London UK; ^3^ The RESILIENT Study Patient & Public Involvement & Engagement Advisory Group UK; ^4^ Cysters Charity Birmingham UK; ^5^ The Dartmouth Institute for Health Policy & Clinical Practice, Geisel School of Medicine Dartmouth College Hanover New Hampshire USA; ^6^ The RESILIENT Study Group UK; ^7^ Department of Psychology, Institute of Population Health University of Liverpool Liverpool UK

**Keywords:** childbirth, COVID‐19, healthcare professionals, in‐depth interviews, maternity care, partners, policymakers, pregnancy, qualitative research, women

## Abstract

**Introduction:**

We aimed to explore the conceptualization and perception of self‐monitoring amongst women, partners, healthcare professionals (HCPs), and policymakers, with particular interest in those living with social/medical complexity.

**Material and Methods:**

Across the United Kingdom, 96 semi‐structured in‐depth qualitative interviews were conducted with 40 women, 15 partners, 21 HCPs, and 20 policymakers to discuss their lived experience of utilizing, delivering, or developing policy for self‐monitoring during the COVID‐19 pandemic. A thematic framework analysis was undertaken to develop themes, considered by participant type, ethnicity, geographical region, personal experience of self‐monitoring, and social complexity, and a content analysis was used to explore how self‐monitoring was conceptualized.

**Results:**

Two themes (and ten sub‐themes) were derived from the Thematic Framework Analysis: “Organizational logistics” (reported by up to 10% participants; sub‐themes: useful resources and infrastructure, lack of instructions and information provided, communication between HCPs and service users, logistical issues, legitimate concerns about clinical practice, and personalization of care) and “Agency and responsibility over care” (reported by up to 6% participants; sub‐themes: anxiety and overwhelm, control over care, avoiding hospitals, and disengaged users). A *post hoc* Qualitative Content Analysis was conducted in a deviation from the protocol which showed women and partners conceptualized self‐monitoring as a general awareness of one's body and monitoring for specific clinical signs, whereas HCPs and policymakers understood self‐monitoring as the use of a device for self‐measurement.

**Conclusions:**

Marked differences exist in how self‐monitoring is conceptualized by service users and service providers, which could influence how service users engage with the practice. Outstanding concerns about implementation include instructions for service users, communication between service users and service providers, HCP workload, safety and quality of care, and the management of disengaged users when self‐monitoring is used to replace care delivered face to face.

## Graphical abstract

We found a dissonance between how self‐monitoring is conceptualised by women, partners, healthcare professionals, and policy‐makers. This disconnect may impact experiences and engagement with care, particularly for those with medical or social complexity. Concerns were raised about safety, communication, care personalisation, and risk of disengagement.

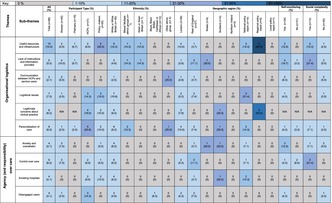

**Abbreviations**
ABPM: Ambulatory blood pressure monitoring
BP: blood pressure
CRN: Clinical Research Network
GDM: gestational diabetes mellitus
HCP: healthcare professional
IDI: In‐depth interview
IMD: Index of multiple deprivation
NHS: National Health Service
NIHR: National Institute of Health and Care Research
RCOG: Royal College of Obstetricians and Gynecologists
SDoH: Social determinants of health
SES: socioeconomic status
SMBP: Self‐monitoring of blood pressure
UK: United Kingdom

**Key message**

We found a dissonance between how self‐monitoring is conceptualized by women, partners, healthcare professionals, and policymakers. This disconnect may impact experiences and engagement with care, particularly for those with medical or social complexity. Concerns were raised about safety, communication, personalization of care, and the risk of disengagement.

## 1 INTRODUCTION

In response to the recent global pandemic (11 March 2020 to 5 May 2023),^1^ strict lockdown restrictions alongside social and physical distancing measures were put in place within the UK National Health Service (NHS). Such rapid reconfiguration of services was undertaken to facilitate the provision of high‐quality maternity care while minimizing the communicable disease risk associated with travel and in‐person contact. The Royal College of Obstetricians and Gynecologists (RCOG) published frequent and detailed guidance based on available evidence,^2^ to enable individual Hospital Trusts to implement self‐monitoring. This included the management and distribution of blood pressure measurement devices purchased by NHS England and NHS Improvement, along with actions for women based on recorded values,^2^ with similar remote measurement, recording, and communication of blood sugar levels for women with gestational diabetes mellitus (GDM).

Self‐monitoring in addition to usual maternity care has been evaluated for the two most common pregnancy complications: hypertension (up to 10% of pregnancies) and GDM (up to 8% of pregnancies),^3^ conditions which disproportionately affect those from ethnic minority groups.^4^, ^5^, ^6^ Self‐monitoring of blood pressure (BP) in hypertensive disorders or blood glucose in GDM is acceptable to women and birthing people, as well as to healthcare professionals (HCPs).^6^, ^7^, ^8^, ^9^, ^10^ Those who monitor their own BP report high motivation and empowerment, greater knowledge, and greater control over their maternity care.^7^, ^8^ Although HCPs find the practice to be acceptable, they vary in the confidence they place in at‐home readings.^7^, ^8^ For the management of GDM, the use of apps facilitating two‐way communication between service users and HCPs (vs. usual care) decreases glycemic index^9^, ^10^ and is preferred by women to avoid multiple hospital visits and long waiting times.^11^ Nevertheless, there is still limited research on the experiences of different stakeholder groups, which utilized or supported self‐monitoring in the UK maternity services during the recent pandemic.^12^, ^13^

This analysis was a component of the qualitative arm of the RESILIENT programme: “Post‐pandemic planning for maternity care for local, regional, and national maternity systems across the four nations”.^14^ We aimed to explore the meaning of self‐monitoring, as well as the lived experiences of undertaking or facilitating self‐monitoring during pregnancy, from the perspectives of: women; fathers, partners, and non‐gestational parents (referred to as “partners”); HCPs; and policymakers, with a specific emphasis on hearing from those in ethnic minority communities and/or those experiencing social or medical complexity.

## 2 MATERIAL AND METHODS

### 2.1 Research governance

A study protocol for RESILIENT has been published,^15^ outlining the full details of the qualitative methodologies employed for analyses of these data.

The RESILIENT Study was funded by the National Institute for Health and Care Research Health Services & Delivery Research Programme (ref:‐NIHR13429). The funder had no role in the study design, recruitment, data collection, analysis, interpretation of the data, or in the writing of the report or decision to submit the paper for publication. Whilst all authors had access to the dataset, a smaller group [TD/GH/HDM/HB] was responsible for analyzing the data, overseen by more senior researchers [SAS/AE/LAM]. All authors were involved in the decision to submit the manuscript, with the first and senior authors [TD/LAM/SAS] leading on the submission.

The RESILIENT Study had a Patient and Public Involvement and Engagement Advisory Group (PPIE‐AG) of 15 members, and a Technical Advisory Group (TAG) of 19 members, both from across the UK.

### 2.2 Recruitment and participants

We had expected to recruit 100 people to interview in this study, with the emphasis on women's interviews. We recruited fewer HCPs than expected, often due to their clinical commitments during the pandemic; but more policymakers than expected, often due to their interest in their voices being heard in the study. As partners are usually difficult to recruit in maternity research, we expected this number to be smaller than that of women recruited, in the same timeframe of recruitment.

Semistructured in‐depth interviews (IDIs; *N* = 96) were conducted with women (*n* = 40), partners (*n* = 15), HCPs (*n* = 21), and policymakers (*n* = 20) between May 2022 and February 2023. Participants were asked about their lived experiences of utilizing, delivering, or developing policy related to self‐monitoring of symptoms in pregnancy during the COVID‐19 pandemic.

Age ranged between 23 and 70 years and 68% of participants identified as White or White British, 7% as Asian or Asian British, 13% as Black or Black British, 7% as Mixed or multiple ethnicities, 4% as any other ethnicity, and 1% preferred not to disclose their ethnicity. For women, partners, and HCPs, 50% utilized or delivered maternity services in London, 36% across the rest of England, 5% in Wales, 5% in Scotland, and 4% in Northern Ireland. Policymakers were categorized on the scope of their policy‐related work, with 70% exercising national reach, 25% having regional reach, and 5% having only local reach. Amongst women and partners, 25% reported at least one aspect of social complexity, and 44% reported medical complexity, which required self‐monitoring. Social complexity was self‐identified by participants and included: lack of social support, mental health problems, or belonging to a minority group relating to sexual orientation or gender identity. Medical complexity was defined as having had to perform self‐monitoring of symptoms during pregnancy for any complication, including: hypertension, gestational diabetes, additional scans for predisposition to genetic complications, or previous pregnancy loss. Additionally, we collected information on deprivation level (as per the Index of Multiple Deprivation)^16^; gender and sexual orientation; care team (for women and partners); and profession, current role, and years in role (for HCPs and policymakers). These findings are presented in Table 1.

**TABLE 1:** Demographics of interview participants.

	Women *N* = 40 (%)	Partners *N* = 15 (%)	HCPs *N* = 21 (%)	Policy‐ makers *N* = 20 (%)	Total *N* = 96 (%)
Age at interview					
18–25	2 (5)	0 (0)	0 (0)	0 (0)	2 (2.1)
26–30	5 (12.5)	6 (40)	1 (4.8)	0 (0)	12 (12.5)
31–40	28 (70)	7 (46.7)	5 (23.8)	1 (5)	41 (42.7)
41–50	5 (12.5)	2 (13.3)	4 (19)	5 (25)	16 (16.7)
51–60	0 (0)	0 (0)	8 (38.1)	8 (40)	16 (16.7)
61–70	0 (0)	0 (0)	2 (9.5)	3 (15)	5 (5.2)
Prefer not to say	0 (0)	0 (0)	1 (4.8)	3 (15)	4 (4.2)
Ethnicity					
White/White British	24 (60)	7 (46.7)	17 (81)	17 (85)	65 (67.8)
English/Welsh/Scottish/Northern Irish/British	19	5	12	13	49
Irish	1	0	1	1	3
Any other White background	4	2	4	3	13
Asian/Asian British	6 (15)	0 (0)	0 (0)	1 (5)	7 (7.3)
Any other Asian background	0	0	0	1	1
Bangladeshi	2	0	0	0	2
Chinese	1	0	0	0	1
Indian	3	0	0	0	3
Black/Black British	6 (15)	4 (26.7)	1 (4.8)	1 (5)	12 (12.5)
African	1	2	0	0	3
Any other Black/African/Caribbean background	2	1	1	0	4
Caribbean	3	1	0	1	5
Mixed/Multiple ethnicity	2 (5)	4 (26.7)	1 (4.8)	0 (0)	7 (7.3)
Any other Mixed/ Multiple ethnic background	0	0	1	0	1
White and Asian	1	1	0	0	2
White and Black African	0	1	0	0	1
White and Black Caribbean	1	2	0	0	3
Other ethnic group	2 (5)	0 (0)	2 (9.5)	0 ()	4 (4.2)
Any other ethnic group	1	0	1	0	2
Arab	1	0	1	0	2
Prefer not to say	0 (0)	0 (0)	0 (0)	1 (5)	1 (1.0)
Geographic location					
London	18 (45)	8 (53.3)	12 (57.1)	n/a	38 (50)
East of England	2 (5)	1 (6.7)	2 (9.5)	n/a	5 (6.6)
Midlands	3 (7.5)	0 (0)	2 (9.5)	n/a	5 (6.6)
North East	4 (10)	2 13.3	1 (4.8)	n/a	7 (9.2)
North West	3 (7.5)	1 (6.7)	0 (0)	n/a	4 (5.3)
South East	2 (5)	0 (0)	0 (0)	n/a	2 (2.6)
South West	2 (5)	1 (6.7)	1 (4.8)	n/a	4 (5.3)
Wales	3 (7.5)	0 (0)	1 (4.8)	n/a	4 (5.3)
Scotland	2 (5)	1 (6.7)	1 (4.8)	n/a	4 (5.3)
Northern Ireland	1 (2.5)	1 (6.7)	1 (4.8)	n/a	3 (4)
National reach (policymakers)^a^	n/a	n/a	n/a	14 (70)	14 (70)
Regional reach (policymakers)^a^	n/a	n/a	n/a	5 (25)	5 (25)
Local reach (policymakers)^a^	n/a	n/a	n/a	1 (5)	1 (5)
Index of multiple deprivation					
1 (most deprived)	3 (7.5)	0 (0)	0 (0)	0 (0)	3 (3.1)
2	3 (7.5)	1 (6.7)	0 (0)	0 (0)	4 (4.2)
3	2 (5)	0 (0)	1 (4.8)	3 (15)	6 (6.3)
4	4 (10)	1 (6.7)	1 (4.8)	1 (5)	7 (7.3)
5	5 (12.5)	5 (33.3)	3 (14.3)	1 (5)	14 (14.6)
6	5 (12.5)	0 (0)	2 (9.5)	1 (5)	8 (8.3)
7	4 (10)	4 (26.7)	4 (19)	2 (10)	14 (14.6)
8	6 (15)	3 (20)	3 (14.3)	0 (0)	12 (12.5)
9	5 (12.5)	0 (0)	0 (0)	4 (20)	9 (9.4)
10 (least deprived)	3 (7.5)	0 (0)	2 (9.5)	1 (5)	6 (6.3)
Prefer not to say	0 (0)	1 (6.7)	5 (23.8)	7 (35)	13 (13.5)
Social complexity (only women and partners)					
Yes	14 (35)	0 (0)	n/a	n/a	14 (25.5)
No	25 (62.5)	15 (100)	n/a	n/a	40 (97.6)
Prefer not to say	1 (2.5)	0 (0)	n/a	n/a	1 (2.4)
Self‐monitoring of symptoms (only women and partners)					
Yes	20 (50)	4 (26.7)	n/a	n/a	24 (43.6)
No	20 (50)	11 (73.3)	n/a	n/a	31 (56.4)
Professional background (only HCPs and policymakers)					
Midwifery	n/a	n/a	5 (23.8)	9 (45)	14 (34.1)
Obstetrics	n/a	n/a	10 (47.6)	5 (25)	15 (36.5)
Neonatologist	n/a	n/a	0 (0)	2 (10)	1 (2.4)
Nursing/health visiting	n/a	n/a	2 (9.5)	2 (10)	3 (7.3)
Pediatric nursing	n/a	n/a	1 (4.8)	0 (0)	1 (2.4)
Other clinical specialty	n/a	n/a	2 (9.5)	0 (0)	2 (4.9)
Policy/civil service	n/a	n/a	1 (4.8)	3 (15)	4 (9.8)
Nonclinical research	n/a	n/a	0 (0)	1 (5)	1 (2.4)
Years in current role (only HCPs and policymakers)					
<2 years	n/a	n/a	3 (14.3)	6 (30)	9 (22)
2–5 years	n/a	n/a	6 (28.5)	10 (50)	16 (39)
6–10 years	n/a	n/a	4 (19)	1 (5)	5 (12)
11+ years	n/a	n/a	8 (38.1)	3 (15)	11 (26.8)

Abbreviation: HCP, healthcare professional.

^a^ For policymakers, data was collected on the geographic scope of their work, rather than physical residence; % reflects the same.

### 2.3 Qualitative Analyses

A Thematic Framework Analysis (TFA)^12^ was utilized, stratifying by participant type, ethnicity, geographic region, presence of self‐monitoring, and presence of social complexity. TFA involves developing a thematic framework, coding the data, charting it into a structured matrix, and finally interpreting patterns within the charted matrix.^12^

The field notes of the primary interviewer (TD), and anecdotal evidence from the IDI analysis, suggested that participants were conceptualizing self‐monitoring differently from care providers. Following discussion with the RESILIENT PPIE‐AG and TAG, the team was encouraged to further interrogate this hypothesis. In a deviation from the protocol,^15^ we conducted an independent, secondary Qualitative Content Analysis (QCA).^13^ Transcripts were interrogated to identify any text related to the monitoring of symptoms, whether at home or with a care provider, to develop inductive categories. This form of QCA is more appropriate when undertaking exploratory analyses and avoids biasing analysis with preconceived or preset codes.^13^ Transcripts were coded to achieve 100% coverage of relevant data from all participants, with codes grouped into clusters, as per guidance for conventional QCA, and frequencies tallied to identify differences between participant groups.

## 3 RESULTS

### 3.1 Thematic Framework Analysis

We identified two themes and 10 subthemes describing the lived experiences and perceptions of the at‐home self‐monitoring of women, partners, HCPs, and policymakers. These are summarized in Table 2, along with the frequency of references for each sub‐theme. Very few subthemes were endorsed by more than ~10% of participants, with more HCPs and policymakers commenting (as compared to women and partners), little contribution from minority ethnic groups, and only one comment from Black/Black British participants.

**TABLE 2:** Participants lived experiences and perceptions of self‐monitoring in pregnancy.

Themes	Subthemes	All (%)	Participant type (%)				Ethnicity (%)					Geographic region (%)								Self‐monitoring (%)		Social complexity (%)	
		Total (*n* = 96)	Women (*n* = 40)	Partners (*n* = 15)	HCPs (*n* = 21)	Policy ‐makers (*n* = 20)	White or White British (*n* = 65)	Mixed/multiple ethnic groups (*n* = 7)	Asian or Asian British (*n* = 7)	Black, Black British, Caribbean or African (*n* = 12)	Other ethnic group (*n* = 4)	London (*n* = 38)	Rest of England (*n* = 27)	Wales (*n* = 4)	Scotland (*n* = 4)	Northern Ireland (*n* = 3)	National policy reach (*n* = 14)	Regional policy reach (*n* = 5)	Local policy reach (*n* = 1)	Yes (*n* = 24)	No (*n* = 31)	Yes (*n* = 14)	No (*n* = 40)
Organizational logistics	Useful resources and infrastructure	10 (10.4)	2 (5.0)	1 (6.7)	2 (9.5)	5 (25.0)	8 (12.3)	1 (14.3)	1 (14.3)	0 (0)	0 (0)	4 (10.0)	1 (3.7)	0 (0)	0 (0)	0 (0)	2 (14.3)	3 (60.0)	0 (0)	3 (12.5)	0 (0)	2 (14.3)	1 (2.5)
	Lack of instructions and information provided	5 (5.2)	4 (10.0)	1 (6.7)	0 (0)	0 (0)	3 (4.6)	1 (14.3)	1 (14.3)	0 (0)	0 (0)	1 (2.6)	3 (11.1)	1 (25.0)	0 (0)	0 (0)	0 (0)	0 (0)	0 (0)	4 (16.7)	1 (3.2)	3 (21.4)	2 (5.0)
	Communication between HCPs and service users	2 (2.1)	2 (5.0)	0 (0)	0 (0)	0 (0)	1 (2.5)	0 (0)	0 (0)	0 (0)	1 (25.0)	1 (2.6)	1 (3.7)	0 (0)	0 (0)	0 (0)	0 (0)	0 (0)	0 (0)	2 (8.3)	0 (0)	0 (0)	2 (5.0)
	Logistical issues	7 (7.3)	1 (2.5)	0 (0)	3 (14.3)	3 (15.0)	7 (10.8)	0 (0)	0 (0)	0 (0)	0 (0)	2 (5.2)	2 (7.4)	0 (0)	0 (0)	0 (0)	3 (21.4)	0 (0)	0 (0)	1 (4.2)	0 (0)	0 (0)	1 (2.5)
	Legitimate concerns about clinical practice	6 (6.2)	N/A	N/A	4 (19.0)	2 (10.0)	6 (9.2)	0 (0)	0 (0)	0 (0)	0 (0)	3 (7.9)	0 (0)	0 (0)	1 (25.0)	0 (0)	0 (0)	2 (40.0)	0 (0)	N/A	N/A	N/A	N/A
	Personalization of care	9 (9.3)	1 (2.5)	1 (2.5)	5 (23.8)	2 (10.0)	6 (9.2)	2 (13.3)	0 (0)	0 (0)	1 (25.0)	4 (10.0)	2 (7.4)	0 (0)	1 (25.0)	0 (0)	2 (14.3)	0 (0)	0 (0)	1 (4.2)	1 (3.2)	1 (7.1)	1 (2.5)
Agency (and responsibility) over care	Anxiety and overwhelm	4 (4.1)	3 (7.5)	0 (0)	0 (0)	1 (5.0)	4 (6.2)	0 (0)	0 (0)	0 (0)	0 (0)	0 (0)	1 (3.7)	1 (25.0)	1 (25.0)	0 (0)	1 (7.1)	0 (0)	0 (0)	3 (12.5)	0 (0)	1 (7.1)	2 (5.0)
	Control over care	6 (6.2)	3 (7.5)	0 (0)	2 (9.5)	1 (5.0)	6 (9.2)	0 (0)	0 (0)	0 (0)	0 (0)	2 (5.2)	3 (11.1)	0 (0)	0 (0)	0 (0)	1 (7.1)	0 (0)	0 (0)	1 (4.2)	2 (6.5)	3 (21.4)	0 (0)
	Avoiding hospitals	4 (4.1)	0 (0)	0 (0)	2 (9.5)	2 (10.0)	4 (6.2)	0 (0)	0 (0)	0 (0)	0 (0)	0 (0)	1 (3.7)	0 (0)	1 (25.0)	0 (0)	2 (14.3)	0 (0)	0 (0)	0 (0)	0 (0)	0 (0)	0 (0)
	Disengaged users	4 (4.1)	1 (2.5)	0 (0)	3 (14.3)	0 (0)	3 (4.6)	0 (0)	0 (0)	1 (8.3)	0 (0)	2 (5.2)	2 (7.4)	0 (0)	0 (0)	0 (0)	0 (0)	0 (0)	0 (0)	1 (4.2)	0 (0)	0 (0)	1 (2.5)

Key	0%	1%–10%	11%–20%	21%–30%	31%–50%	51%–100%

Tables 3 and 4 present supporting participant quotations for Themes 1 and 2, respectively. Participant quotations from women are abbreviated as *W*, partners as *P*, HCPs as *H*, and policymakers as *M*.

**TABLE 3:** Theme 1: Organizational logistics, subthemes, and representative quotations.

Subthemes	Quotations
Useful resources and infrastructure	They sent me an email with a video explaining how to do it, so that was much more helpful than a list of instructions. It was much more helpful to see it visually. It was quite easy to remember when to do it. You just do it when you wake up and then after every meal. There was an app, I think. Yes, there was an app that I used and it was very easy to record so, yes, it was in terms of recording the sugars, yes, it was relatively simple.—W031 We already had apps set up for people to do their blood sugar monitoring and blood pressure monitoring, pre the pandemic, anyway. That was something we were offering women. And the other thing was, we already had digital records in place, which again, I was really pleased that we'd done that. It had been in place for a year, I think, prior to the pandemic and I mean, that enabled us to run a virtual service because obviously we had visibility of the woman's records without having to have her there with us.—M005
Lack of instructions and information provided	But make sure you do all of it. So, I would say, if it wasn't for the fact that we had done it already, it just felt like it was a rush job, in terms of, “There is everything. See you later, good luck.”—P011. The only thing I didn't like was, I was given a figure at the beginning to say, “We need to get your blood sugars below this level.” And then once I got there, the goalpost was moved. I'd rather have been told the final figure upfront, instead of feeling like the goalpost is being moved.—W035
Communication between HCPs and service users	I now recognize as quite poor diabetes care; I should have been having regular visits and conversations, I'd have a 20‐min phone‐call maybe every other week with whomever, it was different people every week. I know the initial consultation was in person, but that was because I'd requested it because they were planning on sending me all of my insulin and needles through the post, basically, that's what they wanted to do and I went “No, you can't do that, I haven't got a clue what you're going on about…”—W027
Logistical issues	I think our consultations potentially took longer because GDm‐Health I think increases work, it doesn't decrease it, because you have glucose values that you can see all the time. So if you check the system again, half an hour after you checked it, there will be new alerts and there will be new things that you have to manage. So it was really learning how to channel our energies in a way that was acceptable… Quality of the data—the same thing. You would have had women before who would have manually written in the same number across the whole day. Some women are really good at uploading their results, some women are really bad.—H015
Legitimate concerns about clinical practice	I know that a lot of the appointments for women were cutting down into telephone appointments and that's just not good enough, I don't think. When you are assessing women antenatally you want to check their blood pressure and their urine and if they've got any concerns and all that sort of thing and I just think a quick 5 min on the telephone isn't going to do that, so I don't think it… While I think it can work in some cases, I think you do risk missing things and I think… well if we look at the evidence, we have missed things.—H021
Personalization of care	I think it can work very well for some people, some people who were able to do that, who understand what they're doing, who can do it accurately, and a lot of people can. Lots of people who have chronic conditions like diabetics are really, really good at it. They do it all the time. So, there's no reason why it couldn't work. But it has to be tailored, so it has to only be done by people who want to do it. It mustn't be forced on people who don't want to do it or don't feel comfortable doing it. And it shouldn't be used as an alternative if we're short staffed, because that means it ends up being done just for the sake of expediency and efficiency and not for the sake of clinical quality.—M001

*Note*: W women; *P* partners; H: HCP; M: policymaker.

**TABLE 4:** Theme 2: Agency (and responsibility) over care, subthemes, and representative quotations.

Subthemes	Quotations
Anxiety and overwhelm	They did recommend I got a blood pressure monitor at one point. I said I'd rather not, because I do struggle a little bit with anxiety, and I thought if I measure it, and it's a little bit higher than it was last time, I'm going to keep checking it, and it's going to keep going up, because I'll get more and more worried about it.—W024
Control over care	I'm quite happy to do all the readings and everything myself, that was not an issue. I don't think there's really any other way of doing that for gestational diabetes, I think it's the most sensible way to do it. Because you've got more control over what you do at home then. If you can see your blood sugars going up, you can do something to counteract it.—W035
Avoiding hospitals	Even though people recognized that maybe something wasn't right, they didn't contact the maternity services because they were scared to come to the maternity service because the issue that they had wasn't as bad as them potentially getting Covid if they came in and so that was probably one of the challenges was how we could encourage people to say you need to come in. And I think in the early days it was particularly difficult when they couldn't have their partners come in with them. People didn't want to come in because they couldn't bring their partner and so again they avoided coming in. That was just an issue.—M019
Disengaged users	So the women I worry about are the women who just don't take their readings because if you don't take your readings for a week, but you come and see someone once a week and we at least get one reading and some idea and can check up on you, that's something, but women who just don't prioritize their own health fell through the cracks.—H019

*Note*: W: women; *P*: partners; H: HCP; M: policymaker.

#### 3.1.1 Organizational logistics

This first theme comprised six sub‐themes: (1) Useful resources and infrastructure, (2) Lack of instructions and information provided, (3) Communication between HCPs and service users, (4) Logistical issues, (5) Legitimate concerns about clinical practice, and (6) Personalization of care.

Positive experiences related to “Useful resources and infrastructure” were reported primarily by policymakers (*n* = 5; 25%), specifically those with regional responsibility (*n* = 3; 60%). Examples included audio‐visual instructions, easy‐to‐use app interfaces, flexibility in working from home, and improved infrastructure and funding for digital technologies. On the other hand, women more often reported on “Lack of instructions and information provided” (*n* = 4; 10%), stating that they often had to rely on unofficial sources; this was highlighted in particular by participants from Wales (*n* = 1; 25%) and those living with social complexity (*n* = 3; 21.4%). Further, a small number of individuals reported poor “Communication between HCPs and service users,” such as between different care teams and for management of multiple complications, though no particular pattern could be identified with respect to participant group characteristics; although there were no comments by service providers “Logistical issues” was most often from the HCP (*n* = 3; 14.3%) or policymaker (*n* = 3; 15%) perspective, including measuring kits being unreliable, questioning the accuracy and validity of self‐measurements, or technology problems. Similarly, HCPs (*n* = 4; 19%) and policymakers (*n* = 2; 10%) also raised “legitimate concerns about clinical practice,” especially regarding increased workload and not being able to assess medical conditions fully via telephone appointments. Participants from all groups emphasized the need to adapt management of self‐monitoring to individual needs, such as social or medical complexity, or English language skills, therefore increasing the need for “personalization of care,” but analysis showed no clear pattern of origin for these comments.

#### 3.1.2 Agency (and responsibility) over care

This second theme comprised four sub‐themes: (1) Anxiety and overwhelm, (2) Control over care, (3) Avoiding hospitals, and (4) Disengaged users.

Feelings of “anxiety and overwhelm” were reported by participants, due to either too much or too little contact with HCPs, particularly for those performing self‐monitoring (*n* = 3; 12.5%) or living with social complexity (*n* = 1; 7.1%). Nevertheless, some participants felt self‐monitoring provided increased “control over care,” with HCPs (*n* = 2; 9.5%) feeling better able to pick up complications earlier through access to all digital patient records, and service users with social complexity (*n* = 3; 21.4%) reporting this form of care interaction as favorable. Service providers perceived women's benefit of avoiding risk of infection by “voiding hospitals,” and appreciated being in the comfort of their own homes, but no service users endorsed this sub‐theme. No clear pattern was evident, bar a slightly increased endorsement by policymakers with national reach (*n* = 2; 14.3%). Finally, HCPs (*n* = 3; 4.3%) discussed difficulty with “disengaged users,” who are not interested in participating in their care for a multitude of reasons.

### 3.2 Protocol‐Driven Analysis versus Data‐Driven Analysis

The lack of reporting on self‐monitoring, despite direct questioning in the RESILIENT in‐depth interviews, was striking given the volume of responses to direct questioning about vaccination, as presented in our other work.^17^ To gain a better understanding of self‐monitoring and how it was conceptualized by the different participant groups, we undertook a *post hoc* QCA.

### 3.3 Conventional Qualitative Content Analysis

Data from the 96 interviews were coded into five main categories. Three could be clustered together as “Meaningfully Conceptualising Self‐Monitoring,” namely: (1) General Awareness of one's body, (2) Self‐motivated to monitor clinical signs and symptoms, and (3) Instructed by HCP to monitor symptoms with a device. The additional two categories were clustered into “Absence and/or Avoidance of Self‐Monitoring,” viz: (4) In‐hospital monitoring, and (5) No monitoring. A description and frequency of each cluster and category is presented in Table 5. Participant quotations from women are abbreviated as *W*, partners as *P*, HCPs as *H*, and policymakers as *M*.

**TABLE 5:** Content analysis frequency table and supportive quotations.

Coding cluster	Coding categories (type of monitoring)	Description	Participant type	Excerpts coded *N* (%)	Supportive quotations
Meaningfully conceptualizing self‐monitoring	General awareness of one's body	*Kept up with changes in the body during pregnancy, checking for (reduced) movements, scar healing* etc.	Women	11 (84.6)	Yes, she did some check‐up assignments like checking her weight, checking the baby's weight, blood. So yes, she definitely did some self‐check‐up.—P012 I think I was just looking out for the same thing that had happened last time [with a miscarriage]. But I don't think there was any advice on that, yes.—W002
			Partners	2 (15.4)	
			HCPs	0 (0.0)	
			Policymakers	0 (0.0)	
	Self‐motivated to monitor clinical signs and symptoms	*Monitored clinical symptoms for own self, not because HCP instructed to do for management of a complication*	Women	12 (92.3)	I monitored my own blood pressure because at times it would get a little high, so I kept an eye on that. And I'd come off a lot of my fibro drugs when we were trying to get pregnant, I'd gone back onto one which was a fibro drug and it's also an antidepressant, so when I came off that, I was in contact with my GP more, monitoring my systems of coming off an antidepressant because obviously that can be tricky. So I do monitor my pain and so on, so there's that but nothing else. And I just kept a log of anything and if ever I was concerned, so about, say, small amounts of bleeding, about my blood pressure, I could ring the midwifery team. I had a mobile for my named midwife and I could leave a message and they'd get back to me.—W009 None of the midwives were coming into the room, that she was having to monitor everything, and keep an eye on the baby's heartbeat. Then she had to escalate it, because my daughter's heartrate was dropping. We then sped things up along. Now, if my wife wasn't really clued onto that, then something dangerous could have happened, both to herself and the baby.—P013
			Partners	1 (7.7)	
			HCPs	0 (0.0)	
			Policymakers	0 (0.0)	
	Instructed by HCP to monitor symptoms with a device	*Those who were told to measure (*e.g., *blood pressure or glucose) at home using a device, at specific times, following instructions*	Women	11 (22.9)	For gestational diabetes, I think they were doing a trial study whereby I had to text in my numbers regularly, so I'd get a morning and evening message reminding me. They got those numbers, it went to their system, but regardless of that, any time I had a number that was above the normal range, I still had to call the line and I would call them, and then leave a message, and they'd call me back and see all the numbers.—W013 So we did a lot of the blood pressure monitoring, the diabetes monitoring, and actually, with the blood pressure monitoring, we were ahead of our time, so we were already doing that, even before the pandemic. But obviously the pandemic came with funding, and that gave us lots of blood pressure machines that we give to our patients, for example to monitor for preeclampsia. Diabetes has always been self‐monitoring, and we have an app where the results come to that.—H007 I think it can work very well for some people, some people who were able to do that, who understand what they're doing, who can do it accurately, and a lot of people can. Lots of people who have chronic conditions like diabetics are really, really good at it. They do it all the time.—M001 So I think she ended up getting tested for that, for gestational diabetes, and I think she had to monitor herself at home, I think.—P009
			Partners	3 (6.3)	
			HCPs	15 (31.3)	
			Policymakers	16 (33.3)	
Absence and/or avoidance of self‐monitoring	In‐hospital monitoring	*Responses related to going into hospital for additional checks/ measurements or HCP visits at home*	Women	8 (88.9)	It was a pregnancy that I was getting told that my baby was too small, I was having scans every couple of weeks, there were periods of time when I was unsure if she was moving so because of the complexity that was there, they were wanting to measure and scan.—W027 It was only when she'd had a blood test and the doctor went, “Oh, you've got this. You got Graves'.” “What?” No idea. No history. No prior thing. And that may have contributed to the prior miscarriages because that is one of the complications. So, in terms of self‐monitoring, that was largely having blood tests to make sure of her kidney levels.—P007
			Partners	1 (11.1)	
			HCPs	0 (0.0)	
			Policymakers	0 (0.0)	
	No monitoring	*Did not have to do any self‐monitoring and responded “no” to interview question*	Women	10 (76.9)	No, nothing like that.—P014 No, no, I didn't have anything, no. I was classed as high‐risk, just because I'd been previous emergency caesarean, but no, nothing.—W008
			Partners	3 (23.1)	
			HCPs	0 (0.0)	
			Policymakers	0 (0.0)	

Within the first cluster: “Meaningfully Conceptualising Self‐Monitoring,” participants suggested how self‐monitoring could be conceptualized.

Having a “general awareness of one's body” (e.g. identifying and tracking the changes to the body during pregnancy and ensuring they attended to fetal movement antenatally, and scar healing postnatally) was not so much self‐monitoring in a clinical sense, but a form of interoception. This category was only endorsed by women (*n* = 11/40) and partners (*n* = 2/15); and was not considered at all by either HCPs or policymakers.

The second category explained how people needed to be or were “self‐motivated to monitor clinical signs/symptoms” (e.g. they monitored clinical symptoms for themselves and not just because they had been directed to for management of an ongoing complication). This can be interpreted as closer to a more familiar clinical–academic sense of the concept of self‐monitoring but shifts the locus of control for symptom management exclusively to the patient, rather than engaging in shared decision‐making about the condition and its management. This category was, again, only endorsed by women (*n* = 12/40) and partners (*n* = 1/15); and was not considered at all by either HCPs or policymakers.

The third category within this cluster demonstrated self‐monitoring as only conducted because of having been “instructed by HCP to monitor symptoms with a device”; for example, hypertension and GDM were monitored with a device either bought or provided by the care provider, and measurement of BP and blood glucose was undertaken at home, at set times, following prescribed instructions. This can be interpreted as a heavily protocolized version of self‐monitoring, and while the locus of control for measurement rests firmly with the patient, they have been engaged with their care providers and are subsequently monitored by them. Endorsement of this category came from all participant groups: women (*n* = 11/40), partners (*n* = 2/15), HCPs (*n* = 15/21), and policymakers (*n =* 16/20), and was the only conceptualization of self‐monitoring to gain widespread consensus.

The second cluster: “Absence and/or Avoidance of Self‐Monitoring” documented the ways in which self‐monitoring was missed or overlooked. Participants discussed instead the notion of “in‐hospital monitoring” (e.g. where monitoring of ongoing conditions and any associated additional checks was seen as the responsibility of the hospital or to be completed in home visits by HCPs). Here, we can interpret an almost complete rejection of obligation for monitoring ongoing conditions, with patients firmly placing the locus of responsibility for it back with the healthcare service, likely with the threat and/or risk of no monitoring occurring being accepted as an outcome. This category garnered the least support overall, with only some endorsement from women (*n* = 8/40) and partners (*n =* 1/15), and none from HCPs and policymakers.

The final category was that of “no monitoring” (e.g. reports of not having had to undertake any self‐monitoring and/or simply responded “no” to the direct interview question). Here, the absence of engagement with the concept of self‐monitoring could suggest an avoidance of it, and absence of it, or perhaps a misunderstanding by women and partners as to what and why self‐monitoring might be required during and after pregnancy. This category was endorsed by a sizeable proportion of women (*n* = 10/40) and partners (*n =* 3/15), but unsurprisingly, did not garner endorsement by HCPs or policymakers.

What we see through the analysis is how differently the concept of self‐monitoring is interpreted by the four participant groups of the study. All HCPs and policymakers only perceived self‐monitoring as service users being instructed to monitor symptoms with a device. However, women and partners had more varied responses, which most commonly perceived self‐monitoring as performing any self‐motivated measurements, including monitoring of symptoms and changes to the pregnant and postpartum body, which would not fall under the usual clinical–academic understanding of self‐monitoring.

## 4 DISCUSSION

Qualitative analysis of in‐depth interviews with a diverse group of 96 women, partners, HCPs, and policymakers revealed self‐monitoring was perceived differently by service users and service providers. Women and partners understood self‐monitoring as a broad concept of self‐motivated surveillance for changes occurring in their bodies, including symptoms. However, HCPs and policymakers interpreted self‐monitoring in the context of using a device for self‐measurement.

Our TFA identified two main themes encapsulating participants' experiences of self‐monitoring during pregnancy and the COVID‐19 pandemic: “Organizational logistics” and “Agency (and responsibility) over care.” Whilst HCPs and policymakers were more likely to comment on self‐monitoring than women and partners, there were many subthemes within each theme, reflecting varied, but not highly‐endorsed, concerns across our stakeholder groups. Those concerns most often related to the practicalities of appropriate instructions, communication, and other logistical issues, as well as the need to personalize care. Remaining themes commented on the shift of agency and responsibility over care to service users, citing mixed feelings of anxiety and overwhelm on the one hand, but more control over care and avoiding infection (at hospitals) on the other hand. All groups acknowledged the challenge of disengaged users. Notable differences between service users and service providers were in the themes of “Communication between HCPs and service users” which no service providers commented on, and in “Avoiding hospitals” which no service users commented on.

A striking finding for us was the relatively few responses from our participants to self‐monitoring, as compared to vaccination^17^ and virtual care during the pandemic, two other concepts about which all interviewees were probed as part of the interview guide for the RESILIENT programme of work. No more than 10/96 respondents commented on self‐monitoring, as compared to 70/96 for vaccination and 33/96 for virtual care.

There is a paucity of published literature on self‐monitoring that replaced aspects of face‐to‐face maternity care during the COVID‐19 pandemic in the UK. In two systematic reviews undertaken by our team, self‐monitoring was discussed in only one of 27 studies of women's experiences^18^ and none of 15 studies of HCPs' experiences. For women, in the only relevant study identified, women appreciated increased control and independence over care but were concerned about implementation, particularly setting up new technology and the infrastructure available to support self‐monitoring.^18^

However, considering studies outside pregnancy, beyond the UK, and not limited to the pandemic, our findings are consistent with other qualitative and mixed‐methods investigations of experiences of self‐monitoring in general. Similar to our work, both service users^19^, ^20^, ^21^, ^22^, ^23^, ^24^ and service providers^19^, ^23^, ^24^ found self‐monitoring to be largely acceptable, but had concerns about implementation, the topic often eliciting more responses from service providers (vs. service users).

Many previous studies of self‐monitoring added to clinical care have echoed our sub‐themes. In a Scottish study, women valued the ease and convenience of digital applications to be an important factor in self‐monitoring, whereas service providers were more concerned with reducing workload, ensuring safety, and promoting flexible working.^19^ Other authors have found HCPs to be concerned about disengaged users,^19^, ^22^ echoing our results of the transfer of the locus of responsibility for care to service users; whilst some service users appreciate this, others may not engage with such an approach or find it overwhelming.^22^ A discourse analysis of online, offline, and unofficial sources of data on self‐monitoring showed a struggle between clinicians' paternalistic discourse and service users' lay discourse, both seeking control.^25^

Official guidance on self‐monitoring during the pandemic was rather vague, such as advice to service users to contact maternity services if something “did not feel right.”^2^ However, our results show that women did not often understand measurement targets, or felt they had not been provided with sufficient information and instructions to assess out‐of‐range readings or causes for concern. Among women, this was the strongest theme in our results, which along with social complexity (a focus in this study and our participant sample), and logistical issues of using new digital applications and infrastructure, may have prevented women from accessing and utilizing maternal health care during the pandemic, in line with the first delay in the “three delays model”—the decision to seek care.^26^

A striking finding in our work is the complete lack of response about self‐monitoring from participants of Black or Black British ethnicity, except for one participant who spoke about disengaged users. This is noteworthy, as both hypertension and GDM are managed with self‐monitoring and are more prevalent among minority ethnic groups,^4^, ^5^, ^6^ making it crucial to understand their perspectives. Also, despite innovative and considered recruitment strategies, we found it difficult to recruit policymakers belonging to different ethnic groups, which speaks to the general lack of ethnic diversity in senior leadership in the NHS.^27^

Finally, we make a contribution to the literature by presenting the dissimilarities in the way service users and service providers conceptualize self‐monitoring, and thereby in their perceptions and actions taken regarding monitoring pregnancy signs and symptoms. Here we have a nuanced notional disentanglement of how self‐monitoring is conceptualized differently, which is important given we had expected there to be a shared and/or at least an agreed understanding of what self‐monitoring does and should entail. Research clarifying the concept of self‐monitoring outside maternity care has found similar attributes, wherein from a patient perspective, self‐monitoring was conceptualized as practicing self‐care monitoring at home, either with or without devices.^28^ However, to the best of our knowledge, this is the first in‐depth qualitative exploration of perceptions of self‐monitoring during pregnancy, particularly self‐monitoring used to replace some aspects of in‐person clinical care during the pandemic.

A key strength of this study is its diverse sample of multiple stakeholders, across the four UK nations, and with a specific focus on marginalized groups, a direct reflection of our sampling framework for recruitment. We interviewed service users of varied age, SES, ethnicity, gender, sexual orientation, medical and social complexity, and care providers with varied professional roles and seniority, considering these characteristics in sub‐group analyses, and importantly, ethnicity, SES, and social complexity separately. This allowed us to see marked differences in perspectives by sub‐group, as have been observed by others.^29^, ^30^ While our study benefits from a multi‐method study design, wherein we were able to show the difference in conceptualization of self‐monitoring between groups, we acknowledge that this could have led to differences in understanding interview questions and, consequently, responses.

Limitations of our study include limited diversity of our policymaker participants despite innovative and considered recruitment strategies; as such, our findings may not reflect the views held by other more marginalized sections of society, but they do, nonetheless, speak to the general lack of ethnic diversity in senior leadership in the NHS.^27^

## 5 CONCLUSION

Our findings highlight the need to clearly define self‐monitoring in clinical or research endeavors. Although many across our stakeholder groups viewed self‐monitoring integrated into routine care (as opposed to added to it) positively, there are outstanding concerns about the practicalities, including instructions for service users and their comfort, communication between service users and providers, HCP workload, safety and quality of care, and the management of disengaged users when self‐monitoring is used to replace care which has been previously delivered face to face.

## AUTHOR CONTRIBUTIONS

Conceptualization: T.D., H.B., A.E., H.D.M., A.D.V.C., E.C.N., S.A.S., L.A.M.; methodology: T.D., A.E., S.A.S.; software: T.D., S.A.S.; validation: H.B., A.E., K.K., L.M., A.D.V.C., E.C.N., P.v.D., S.A.S., L.A.M.; formal analysis: T.D., G.H., H.B., A.E., H.D.M., A.D.V.C., E.C.N., P.v.D., S.A.S., L.A.M.; investigation: T.D., H.B., S.A.S., L.A.M.; resources: S.A.S., L.A.M.; data curation: T.D., S.A.S., G.H., H.D.M., P.v.D., L.A.M.; writing—original draft: T.D., S.A.S., L.A.M.; writing—review and editing: H.B., A.E., K.K., L.M., H.D.M., G.H., A.D.V.C., E.C.N., P.v.D., The RESILIENT Study Group, S.A.S., L.A.M.; visualization: T.D., H.D.M., G.H., P.v.D., S.A.S., L.A.M.; supervision: S.A.S., A.E., L.A.M.; project administration: T.D., H.D.M., G.H., S.A.S., L.A.M.; funding acquisition: H.B., A.E., A.D.V.C., E.C.N., P.v.D., S.A.S., L.A.M.

## FUNDING INFORMATION

The RESILIENT Study was funded by the National Institute for Health and Care Research Health Services & Delivery Research programme (ref: NIHR134293) awarded to Laura A. Magee, Sergio A. Silverio, Harriet Boulding, Abigail Easter, Aricca D. Van Citters, Eugene C. Nelson, Peter von Dadelszen, and Members of The RESILIENT Study Group. Sergio A. Silverio was in receipt of a Personal Doctoral Fellowship awarded by the National Institute for Health and Care Research Applied Research Collaboration—South London [NIHR ARC‐SL] Capacity Building Theme (ref: NIHR‐INF‐2170). Tisha Dasgupta is in receipt of a Health Practices, Innovation & Implementation [HPII] Doctoral Fellowship (ref: ES/P00703/1) funded by the Economic & Social Research Council [ESRC] as part of the London Interdisciplinary Social Science Doctoral Training Partnership [LISS DTP]. Abigail Easter is supported by the National Institute for Health and Care Research Applied Research Collaboration South London [NIHR ARC‐SL] at King's College Hospital NHS Foundation Trust. The views expressed are those of the authors and not necessarily those of the NIHR or the Department of Health and Social Care. The funders had no role in the work or write‐up associated with this manuscript, and the views expressed are those of the authors and not necessarily those of the funders.

## CONFLICT OF INTEREST STATEMENT

The RESILIENT Study has been adopted and is supported by the National Institute for Health and Care Research Applied Research Collaboration South London [NIHR ARC South London] at King's College Hospital NHS Foundation Trust. The views expressed are those of the authors and not necessarily those of the NIHR or the Department of Health and Social Care. The authors have no conflicts of interest to declare.

## ETHICS STATEMENT

The qualitative work for The RESILIENT study was approved by the King’s College London Health Faculties Research Ethics Subcommittee (HR/DP‐21/22‐26740) on February 21, 2022.

## Data Availability

The data that support the findings of this study are available on request from the corresponding author. The data are not publicly available due to privacy or ethical restrictions.
